# Chemically Mediated Plant–Plant Interactions: Allelopathy and Allelobiosis

**DOI:** 10.3390/plants13050626

**Published:** 2024-02-24

**Authors:** Chui-Hua Kong, Zheng Li, Feng-Li Li, Xin-Xin Xia, Peng Wang

**Affiliations:** 1 College of Resources and Environmental Sciences, China Agricultural University, Beijing 100193, China; lizheng268@sina.com (Z.L.); lifengli789@163.com (F.-L.L.); 13996772329@163.com (X.-X.X.); 2 Institute of Applied Ecology, Chinese Academy of Sciences, Shenyang 110016, China; wangpeng@iae.ac.cn

**Keywords:** allelochemical responses, chemical signals, neighbor identity recognition, molecular mechanisms, plant–plant signaling interaction, root exudates, root–soil interactions, volatiles

## Abstract

Plant–plant interactions are a central driver for plant coexistence and community assembly. Chemically mediated plant–plant interactions are represented by allelopathy and allelobiosis. Both allelopathy and allelobiosis are achieved through specialized metabolites (allelochemicals or signaling chemicals) produced and released from neighboring plants. Allelopathy exerts mostly negative effects on the establishment and growth of neighboring plants by allelochemicals, while allelobiosis provides plant neighbor detection and identity recognition mediated by signaling chemicals. Therefore, plants can chemically affect the performance of neighboring plants through the allelopathy and allelobiosis that frequently occur in plant–plant intra-specific and inter-specific interactions. Allelopathy and allelobiosis are two probably inseparable processes that occur together in plant–plant chemical interactions. Here, we comprehensively review allelopathy and allelobiosis in plant–plant interactions, including allelopathy and allelochemicals and their application for sustainable agriculture and forestry, allelobiosis and plant identity recognition, chemically mediated root–soil interactions and plant–soil feedback, and biosynthesis and the molecular mechanisms of allelochemicals and signaling chemicals. Altogether, these efforts provide the recent advancements in the wide field of allelopathy and allelobiosis, and new insights into the chemically mediated plant–plant interactions.

## 1. Introduction

When two or more plants coexist, a series of intra-specific and inter-specific interactions occur either positively or negatively. Productivity and the establishment of mixed-species systems or variety mixtures (single-species) are ultimately the net result of positive and negative interactions among the component species or varieties [1,2]. The interplay and balance between positive and negative interactions are important and recurrent topics in plant ecology. Plant–plant negative interactions are mainly involved in competition, parasitism, and incompatibility, while positive interactions include heterospecific facilitation, conspecific cooperation, and complementarity. These patterns can arise through a variety of ecological mechanisms, such as resource partitioning, niche divergence, kinship strategy, and other species-specific behaviors [3,4,5,6]. The performance and outcomes of plant coexistence and community assembly depend on multiple biotic and abiotic interactions, but the central driver must be plant–plant interactions. 

Plant–plant interactions are mediated physically and chemically. An increasing number of studies have shown that chemically mediated plant–plant interactions are important driving forces for plant coexistence and community assembly [7,8,9]. Chemically mediated plant–plant interactions can be represented by allelopathy and allelobiosis. Allelopathy means that plants produce and release allelochemicals to inhibit the establishment and growth of co-occurring plants [10]. Despite the allelochemical actions that are in some way growth-stimulating, by the mainstream perspective, allelopathy is defined as negative interactions among plant species because the term is based on the Greek terms allelo, meaning ‘one another’, and path, for ‘suffering’ [11], while allelobiosis is a relatively new term and consists of allelo and biosis, meaning ‘mutualism’, denoting positive or neutral interactions [12,13]. Both allelopathy and allelobiosis are ecological phenomena leading to chemical interference among plants. Allelopathy describes that plants produce and release allelochemicals to exert negative effects on neighboring plants. In contrast to negative plant–plant allelopathic interactions, allelobiosis describes the transmission of signaling chemicals in plant–plant interactions where the response of the coexisting plants affects their growth and defense strategy and is related to herbivores and their natural enemies, resulting in positive or neutral effects [14,15]. Importantly, allelobiosis is involved in chemical-mediated plant neighbor detection and identity recognition [16]. Accordingly, allelopathy and allelobiosis cover any positive or negative effects on chemically mediated interactions among plants.

**Figure 1:**
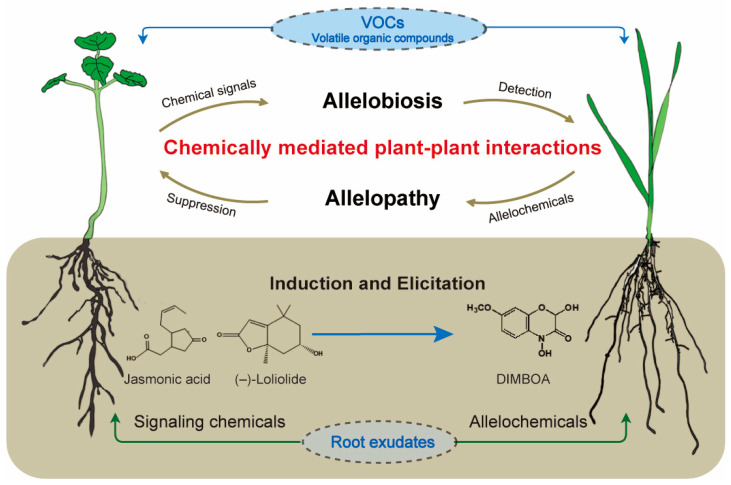
Allelopathy and allelobiosis in plant–plant interactions. VOCs include volatile allelochemicals, such as camphor and (–)-thujone, and volatile signaling chemicals, such as ethylene and methyl jasmonate.

The importance of allelopathy and allelobiosis in plant–plant interactions has received a great deal of attention in the past decades [17,18,19,20,21]. However, allelopathy and allelobiosis are usually studied separately, despite their necessary linkage in nature. There is a wealth of information on plant–plant allelopathic interactions in natural and managed ecosystems [17,18,19,20,22] but a lack of information on the role of allelobiosis in plant–plant interactions; the term allelobiosis is sparsely found in the scientific literature. In particular, most of the allelopathy research has primarily focused on plants and their allelochemical interactions but ignored the plant–plant signaling interactions. Actually, for plant coexistence, plants first may detect and potentially recognize their neighbors, and then initiate allelopathic interference to regulate inter-specific or intra-specific interactions [16,23]. Therefore, allelopathy and allelobiosis are two probably inseparable processes that occur together in plant–plant chemical interactions (Figure 1).

Allelopathy and allelobiosis have provided fascinating insights into eco-evolutionary relationships and represent potential strategies for sustainable agriculture and forestry [9,16,20,21]. This review will focus on the recent advancements in the wide field of allelopathy and allelobiosis in plant–plant interactions. In particular, this review will extend and concretize the concept of allelobiosis from a useful definition for evaluating neighbor detection and identity recognition in plant–plant signaling interactions. Such information is critical to enhance our understanding of chemically mediated plant–plant interactions driving plant coexistence and community assembly, stimulating additional research into plant–plant interactions.

## 2. Allelopathy, Allelochemicals, and Their Application for Sustainable Agriculture and Forestry

### 2.1. Allelopathy, Competition, and Plant Invasion

Plants may affect the performance of neighboring plants through allelopathy, competition, or both. Both allelopathy and competition are naturally occurring ecological phenomena with a negative effect on neighboring plants. However, allelopathy and competition possess different mechanisms. Allelopathy means that plants produce and release allelochemicals into the environment to decrease neighbor fitness, but competition is imposed by the environment where plants draw from the same resource pools (i.e., neighboring plants deplete water and nutrients and provide shade). In addition, allelopathy is a species-specific behavior, but competition occurs invariably in plant coexistence regardless of plant species. Importantly, competition occurs only during living plant coexistence, but allelopathic potential may have an impact on successive plants by releasing allelochemicals from dead plants and their decomposing residues [11,18]. 

Allelopathy and competition frequently occur in conspecific and heterospecific plant–plant interactions. Intra-specific allelopathy is called autotoxicity; a plant releases allelochemicals to inhibit the growth and establishment of other conspecific plants [24]. Intra-specific competition generally must be stronger than inter-specific competition, where conspecifics interact more intensely because of their similarity [3]. Plant competition for the same resources is demonstrated by the presence of a neighbor reducing fitness, while allelopathy chemically suppresses the growth of neighboring plants, providing a competitive advantage for their own growth. Therefore, allelopathy is a plant chemical trait or behavior to increase competitive ability. Accordingly, it is not realistic to separate resource competition from allelopathy [25]. Although plants affect the performance of neighboring plants through competition and allelopathy in ways that are difficult to separate, the effects of allelopathy and competition on plant performance have been extensively investigated; however, there is a lack of quantitative synthesis [26]. A meta-analysis of 384 studies found that allelopathy reduced plant performance by 25% but the variation in allelopathy was high. In particular, allelopathy became more negative with increasing phylogenetic distance, indicating that allelopathy might contribute to the coexistence of closely related species (i.e., convergence) or dominance of a single species [27].

Allelopathy has had a checkered history. The classical cases are that black walnut (*Juglans nigra*) produced and released a 1,4-naphthoquinone (juglone) to interfere with the growth of understory plants [28] and the allelopathic interference of shrubs with grass through the release of volatile terpenes into southern California coastal grassland [29]. However, the ecological importance of allelopathy was a controversial issue because much of the early work on the preponderance of phenomenological studies was insufficiently rigorous and confirmable. In the early 2000s, allelopathy was firmly back on the center stage in a case study of invasive plants versus their new and old neighbors [30]. This case study neatly avoids the pitfalls of earlier work and provides compelling evidence that allelopathy is a mechanism for an exotic invasion of Eurasian spotted knapweed (*Centaurea maculosa*) in the western United States [31,32]. The allelopathic invasion of spotted knapweed in American grasslands resulted in the novel weapons hypothesis, as many co-occurring native plant species exhibit a lack of evolved tolerance to novel allelochemicals [33]. Subsequently, an increasing number of studies have shown that allelopathy can contribute to the success of alien plants [34,35,36,37]. 

Allelochemicals play a large part in the successful invasion of certain plant species. Generally, allelochemicals of invasive species have low levels when growing with their original neighbors due to long-term mutual adaptation. In the invaded habitats, however, invasive species produce and release allelochemicals at a high level to exert a strongly allelopathic interference on the native species, conferring a competitive advantage to the invasive plants [34,38]. Therefore, the allelopathy of invasive species is conditional, and there is a discrepancy between geographical sites. For example, spotted knapweed exhibited an allelopathic invasion in the western United States but not in eastern American grasslands [39]. Bohemian knotweed seems not to rely heavily on allelopathy for its persistence in invaded sites in the southwest part of Zagreb, Croatia [40]. 

Invasive plants apply an allelopathic impact on native species to be successful in their invaded range. It is also worthwhile to note that native species co-occurring with invaders have evolved a tolerance to allelopathy. A few studies indicated allelopathy and its coevolutionary implications between native neighbors and invasive species [35,36]. For example, co-occurring native *Juncus pallidus* may evolve tolerance to the allelochemicals induced by invasive *Cynara cardunculus*. In particular, allelochemicals induced by *C. cardunculus* exert more suppressive effects on native plants than non-native plants, linking the coevolved tolerance [36]. Although coevolution can promote the long-term coexistence of two competing species, plant invasions offer opportunities to capture the process of coevolution [35]. Therefore, allelopathy-based plant invasion may drive coexistence via coevolution, altering community structure and ecosystem functions. Not only that, allelopathy has broad ecological consequences for natural systems, and is particularly important for species distribution, conditionality of interactions, and maintenance of species diversity [10,17,20,41].

### 2.2. Allelochemical Responses and Interactions

Plants can modulate their morphological and biochemical traits in response to environmental conditions, particularly for the production of secondary metabolites [42,43,44,45]. Allelopathic actions depend on the production and release of allelochemicals, and thus, allelopathy is inevitably regulated by the environmental factors. Numerous studies have shown that allelopathic potential varies with plant growth conditions, and the categories and amounts of allelochemicals can be modulated by natural factors. In particular, biotic and abiotic stressors including herbivores, pathogens, competition, mechanical wounding, drought, nutrient deficiency, salt, and heavy metals can induce the production and release of allelochemicals [46,47,48,49]. Accordingly, allelopathy is not only a competitive trait, but also one induced in response to the identity of the neighboring plants. As such, allelopathy could represent the keystone in the argument that plants do differentially detect and respond to their neighbors [23,50].

Plant–plant allelopathic interactions occur in the presence of conspecific and inter-specific neighbors, where co-occurring plants are prone to competition. Therefore, an allelochemical response to competitors is vital to understand allelopathic interactions. The context-dependent induction of allelopathy in plants under competition has received great attention in recent decades [23,48,51,52]. The ability to induce allelochemical production in the presence of competitors is a natural adaptive strategy so that plants can save costs of metabolite production in the absence of competitors [48,53]. Although competitor-induced allelochemical response is a general phenomenon in plant–plant allelopathic interactions, the phenomenon is density dependent. Allelopathic plant species increase allelochemical concentrations in response to competitors at low or intermediate densities but a high density of competitors may reduce the production of allelochemicals [23,54]. Plants growing with a high density of neighbors likely experience an increase in competition, resulting in a shift from allelochemical defense to growth, i.e., inducible allelopathy to trade off with constitutive growth [55].

DIMBOA (2,4-dihydroxy-7-methoxy-1,4-benzoxazin-3-one) is a dominant allelochemical in cereal (wheat, maize, and rye, but not in rice) against weeds, pathogens, and herbivores [56]. The production of DIMBOA can be induced by a diverse range of weeds [16,57]. To provide a broader context for the competitor-induced allelochemical response, a dataset of wheat DIMBOA concentration was produced in the presence of a total of 100 different plants species at varying densities. The induction of DIMBOA markedly increased in heterospecific combinations relative to DIMBOA production when competing with conspecifics. Significant increases occurred in 38/100 species at a 5:5 proportion, while occurring in 70/100 species at a 5:8 mixture [23]. Therefore, the presence of either conspecific or heterospecifc neighbors could induce the allelochemical response in a density-dependent manner.

Polyacetylenes are putative allelochemicals in invasive species such as *Solidago altissima*. A recent study indicated the inducibility of polyacetylenes in response to competition [58]. The presence of competitors induced polyacetylenes, but the inducibility was observed at an early stage of *S. altissima* invasion. In addition, induction became more prominent under nutrient depletion, where induced polyacetylenes may be effectively suppressed by the growth of competitors [58]. It appeared from the results that allelopathic plant species could respond to competition by inducing allelochemical production, but competition induced allelochemicals in a context-dependent manner, and variation in resource availability may influence plants’ allelopathic capacity.

Allelopathy and allelochemical responses take place both aboveground and belowground. Aboveground allelopathy responses mediated by volatile allelochemicals are well established. This situation is primarily driven by the accessibility of aerial tissues and the availability of reliable techniques to detect and identify volatile allelochemicals [18]. However, it is extremely difficult to unambiguously demonstrate belowground allelopathy and allelochemical interactions. Unlike aboveground processes, the study of belowground allelochemical interactions is hampered by the inability to observe roots [59,60]. Actually, many allelopathic species release allelochemicals through their root systems into the soil, exerting a negative effect on cooccurring species, particularly for hindering the growth of surrounding roots [60,61]. 

Allelochemical interactions at the root level are commonly measured as a change in root biomass. However, a meta-analysis of 61 studies to assess the effect of allelochemicals on root morphology found that the allelochemicals significantly inhibited root length overall but had little effect on root biomass, root surface area, and root volume [62]. Furthermore, allelopathic wheat challenged with 60 other plant species was used to document the combinations of root morphology, revealing root length as a more responsive trait than root biomass in plant–plant allelopathic interactions [63]. Importantly, allelopathic plants release allelochemicals to alter the placement patterns of neighboring roots. Neighboring plants adjust root placement to avoid these allelochemicals. Allelopathic species generally proliferated roots toward the neighboring target species, while most target species showed root avoidance [60,61,63]. If neighboring species do not alter root placement, their roots should be inhibited by root-secreted allelochemicals from allelopathic species. 

Allelopathic species may interfere with cooccurring plants through the induction and release of allelochemicals, resulting in neighbor suppression. However, allelopathic plants usually coexist with a broad spectrum of conspecific and inter-specific neighbors. The growth of some species is inhibited significantly by allelochemicals but that of other species is not. This issue is partly due to the allelochemicals released from the allelopathic species having relative activity on the specificity of neighbors in a dose-dependent manner, or to some cooccurring species being able to resist the allelochemicals, leading to allelochemical adaptation. Therefore, allelopathic effects may result from multiple factors rather than the allelochemicals only.

### 2.3. Allelopathy for Sustainable Agriculture and Forestry

Allelopathy and allelochemicals have important implications and consequences for plant coexistence and community assembly in natural ecosystems. Particularly intriguing are their potential importance and application for sustainable agriculture and forestry. Accordingly, allelopathy has received increasing attention in cropping systems [19,26,57,60,64]. Much effort has focused on allelopathic interference between crop plants and weeds, particularly for deciphering field-based evidence for crop allelopathy in weed regulation. Several practices, including the use of allelopathic plant mulches [65], incorporation into cultural management options [66], natural herbicides [67], and varietal improvement [68], have been used to improve weed management in cropping systems.

Many weeds use an allelopathic mechanism to affect crop production. A heavy infestation of allelopathic weeds in cultivated fields can result in economically destructive effects on the growth and yield of crop plants. For example, the worst weeds, *Ageratum conyzoides* and *Ambrosia trifida*, can infest and colonize quickly into cultivated fields by producing and releasing allelochemicals, resulting in adverse effects on the growth and yield of crop plants [46,69,70]. Attempts to control them by herbicides have met with limited success. However, these allelopathic weeds and their allelochemicals have been incorporated into ecological pest management or employed for biorational pesticides [67,71,72,73], providing an alternative weed control strategy. Allelopathic weeds have threatened crop production. Fortunately, a few crop varieties can synthesize and release their own ‘herbicides’, i.e., allelochemicals against weeds in fields, resulting in a natural interference of crop plants with weeds [66]. Allelopathic crop varieties hold promise for sustainable weed regulation. However, their agronomic characteristics and grain yield do not meet the commercial standards of the crop industry. Using traditional and biotechnological approaches, selected elite allelopathic crop genotypes have enabled breeding efforts to improve weed suppression traits in commercial cultivars. The successful breeding of commercially acceptable allelopathic rice cultivars with high yield and weed suppression has been incorporated into present rice production systems to minimize the amount of herbicide used [68]. 

Allelopathy also offers implications and applications in forestry. Forest regeneration is a critical ecological process that sustains reproduction through the establishment of saplings. However, regeneration failure usually occurs in natural and managed tree systems, particularly in monoculture tree plantations [74,75,76]. Autotoxicity caused by intra-specific allelopathy may be responsible for this problem. Long-term exposure to tree-derived allelochemicals from litter and root exudates may create a barrier effect on the understory-regenerated saplings, resulting in forest regeneration failure. Apart from regeneration failure, autotoxicity also is a major reason for the productivity decline of tree plantations. A tree plantation is an artificial forest where fast-growing tree species are established in a monoculture manner. However, successive rotations of plantations usually cause a replanting problem or soil disease, resulting in a decline in productivity and the loss of biodiversity in plantations [77,78]. 

*Eucalyptus* is one of the most widely planted forestry genera with the replanting problem. Allelochemicals from *Eucalyptus* litter and roots penetrate into the soil, limiting the regeneration of understory plants [79]. Moreover, intra-specific allelopathy is more crucial than resource competition in the replanting problem of *Eucalyptus* plantations [80]. *Juglans* is another widely planted forestry genus. Several species, such as *Juglans nigra* (Black walnut) and *Juglans mandshurica* (*Manchurian walnut*), are commercially planted worldwide. However, the establishment and productivity decline of replanted walnut plantations remain a significant problem. Allelopathy of black walnut through the allelochemical juglone has been observed and investigated for thousands of years [28]. Similarly, autotoxicity is a major reason for the productivity decline of monospecific *Manchurian walnut* plantations [81]. Chinese fir (*Cunninghamia lanceolata*) has been planted for the large-scale production of wood in China. However, regeneration failure and productivity decline have remained critical problems in monospecific plantations. The major driver of the problems is tree-derived allelochemicals and their autotoxicity [75,76]. 

To mitigate and overcome autotoxicity in tree plantations, one promising option involves mixed-species plantations instead of monocultures. The introduction of certain tree species can alleviate the autotoxicity and soil deterioration caused by allelochemicals in plantations [59,81,82]. Mixed-species plantations of *Eucalyptus* with an introduced N-fixing species *Albizia lebbeck* increase productivity and maintain soil fertility [82]. The establishment and productivity of *Manchurian walnut* can be improved in the presence of *Larix gmelini* [81]. A broadleaf species *Michelia macclurei* enhances the growth and regeneration of Chinese fir [59]. One of the explanations for the improvement is that allelochemicals released from an autotoxic tree species may be decreased in the presence of a “good” neighbor like *L. gmelini* and *M. macclurei*. Furthermore, good neighbors not only induce a microbial shift to accelerate the decomposition rates of allelochemicals but also balance the negative effects of root competition [59,83]. These successful mixed-species plantations, based on empirically established traditional practices, reveal many potential advantages and the ecological importance of allelopathy. Understanding allelopathy will be a key step in using ecological approaches to design species mixtures in tree plantations.

## 3. Allelobiosis and Plant Identity Recognition

### 3.1. Allelobiosis and Plant–Plant Signaling Interactions

Plant-to-plant signaling is a key mediator of interactions among plant species. The interpretation of plant-to-plant signaling can help plants adapt to the local environment. In particular, plants can perceive and respond to chemical cues emitted from their neighbors, altering survival and performance and impacting plant coexistence and community assembly [7,9,84]. Plant-emitted chemical cues mediate plant sensing and communication [85], acting as alarm signals to warn neighboring plants of an imminent herbivorous or pathogen attack [86], or serving as an inter- and intra-plant signal for the detection of neighbors, even kin and non-kin individuals within a species [23,87]. Much of the research into chemical-mediated plant–plant signaling interactions has dealt with volatiles induced by herbivory in initiating defensive responses from a tritrophic perspective [88,89,90]. Therefore, allelobiosis was first introduced by Ninkovic as a volatile signal interpretation and its effects were studied on the plant and subsequent trophic levels. The term “allelobiosis” was given three key aspects of the effect: (1) chemical communication between undamaged plants, (2) benefits for the recipient plants, and (3) the recipient plant’s response influencing other trophic levels of organisms [13].

Allelobiosis originated from volatile-mediated aboveground signaling interactions that have been investigated in greater detail [7,89,90]. However, a great deal of recent attention has been paid to root exudate-mediated belowground signaling interactions among plant species [16,84,91,92]. Accordingly, allelobiosis reasonably extends to plant–plant belowground signaling interactions mediated by root-secreted chemical signals. Allelobiosis refers to the communication among plants through phytochemicals, carrying valuable information that transmits signals between adjacent plants, thereby influencing plant growth, defense, and reproduction. However, the use of the term allelobiosis is relatively limited because allelobiosis shares conceptual similarities with plant chemical communication and signal transduction. Plant chemical communication sometimes includes allelochemical interactions. A few allelochemicals, such as benzoxazinoids and momilactones, may also serve both roles of toxic inhibitors and nontoxic signals [93,94]. Whereas allelobiosis places a stronger emphasis on the role of non-toxic signaling chemicals with an informative value for co-occurring plants, highlighting a specific contribution to chemically mediated plant–plant interactions. Accordingly, allelobiosis in the context of plant–plant interactions is orchestrated by signaling chemicals, operating in both aboveground and belowground components, influencing intra-specific and inter-specific dynamics within plant populations.

Plant–plant signaling interactions involve both physical and chemical signals, including light reflection, nutrient availability, and plant-released secondary metabolites, forming a complex and intricate plant social network. These signals trigger a series of plant response strategies, such as shade avoidance, root foraging, and chemical defense [74,84,89,95]. The perception of light signals plays a crucial role in various aspects of plant growth and development, competition, and adaptation to the environment [96,97,98]. Plants perceive light signals through phytochromes, photoreceptors, and UVR8, and shaded plants primarily perceive light in the green and far-red spectra [99,100,101]. This alteration influences the synthesis and transport of auxins, triggering shade responses in plants, such as stem elongation, adjustment of leaf angle, and modulation of flowering time [100,101], and consequently, the outcomes of aboveground interactions among plants.

Plants are surrounded by a diverse array of chemical signals in their growth environment (Table 1). Plants need to discern and capture valuable information from these signals, eliciting specific physiological and biochemical responses. Ethylene was the first gaseous signal within the plants, changing the immediate environment of the producer, its neighbors, and attackers [102]. Subsequently, methyl jasmonate [103,104], methyl salicylate [105], and other volatile organic compounds (VOCs) [106,107,108,109,110,111,112,113] have been identified as chemical signals for inter- and intra-plant communication, mediated through the air. Methyl benzoate, serving as an aboveground chemical signal in plants, is frequently released into the air environment, participating in the information transmission and interaction between plants [114]. Isoprene, β-caryophyllene, trans-β-ocimene, and several terpenes [7,109] also serve as air-borne chemical signals, inducing neighboring plants to develop resistance against pests. Plant-emitted indole induces the production of defensive metabolites in neighboring plants, contributing to information transmission among plants [115]. Such signaling perception-dependent defenses enable plants to more effectively cope with external environmental challenges and enhance their survival.

Compared to air-borne chemical signals, soil-borne chemical signals (Table 1) have faced challenges and limitations due to the complexity, opacity, and difficulties in directly observing and collecting underground secretions imposed by the soil environment [84,92]. This hinders our understanding of the critical component for comprehensive plant–plant interactions and plant survival strategies. In recent years, there has been increasing in-depth research on the identification and functionality of signaling chemicals in the root exudates and soil environment [23,128,129,130]. Root-secreted jasmonic acid and salicylic acid from barnyardgrass can induce the production of rice allelochemicals [119]. Salicylic acid also stimulates glucosinolate in the root exudation of rapeseed [131]. Root-secreted (–)-loliolide, the most ubiquitous lactone, can induce the production of allelochemicals in rice and wheat [23,130]. This ubiquitous (–)-loliolide can act as a general soil-borne signaling chemical common to plants [132,133]. Co-occurring plants utilize root-secreted (–)-loliolide to convey information on neighbor detection and identity recognition. In addition, (–)-loliolide may act as a phytochemical cue to explain the fitness cost of herbicide-resistant weeds by regulating vitality and fecundity, yielding critical insights into plant–plant signaling interactions [134].

Root-secreted chemical signals involve the common mycelial networks (CMNs) of arbuscular mycorrhizal fungi (AMF) and plant roots (Table 1). CMNs can function as conduits, transferring signals between plants. Between-plant signaling mediated by CMNs has a profound impact on plant–plant interactions [135]. Strigolactones, serving as root signals, play a role in recruiting AMF to colonize plant roots [136]. However, these signals can also be intercepted by parasitic plants, facilitating successful parasitism [125,126,135]. Quercetin-3-*O*-rhamnoside attracts rhizobia, inducing the early formation of root nodules [127]. The signaling mechanisms between plants and rhizospheric microorganisms contribute to plant adaptation in complex soil environments, and the promotion of synergistic growth among plants.

### 3.2. Plant Neighbor Detection and Identity Recognition

Due to the sessile growth characteristics, a plant cannot select its living environment and neighbors. Therefore, plant–plant interactions are inherently local in nature and mostly occur among immediate neighbors. From a traditional perspective, plants only passively adapt to the presence of their neighbors. Recent studies have shown that plants also engage in active detection and recognition of neighboring individuals [23,84,137]. Unlike the passive adjustments that plants make to adapt to their environment, the detection of neighbors by plants represents a proactive behavior, actively engaging in communication and interaction with surrounding plants to achieve more efficient resource utilization and survival strategies [8,84]. In particular, plants actively respond to their neighbors by changing their defensive strategy based on the identity of neighbors [44,63]. Such plant neighbor detection and identity recognition are driven by chemical signals [23,84,92,138].

Chemically, plants can modify their environment by releasing secondary metabolites such as root exudates (liquid) or gaseous VOCs [84,139]. Therefore, plants may detect neighbors through plant volatiles as air-borne signals or root exudates as soil-borne signals. These chemical signals drive aboveground and belowground plant–plant signaling interactions (Figure 1). However, most studies on plant neighbor detection and identity recognition have focused on air-borne signals (Table 1). The studies on soil-borne signals are extremely difficult because the transduction of belowground chemical signals requires root–soil interactions. Nevertheless, neighbor detection and identity recognition do occur at the root level in plants [91]. Many studies have shown that allelopathic plants may detect and recognize their neighbors through the root exudates and respond by increasing the allelochemicals [23,51,57,140,141]. However, most studies did not clarify which individual constituents in the root exudates were responsible for the neighbor detection and allelochemical response. 

To unambiguously demonstrate plant neighbor detection and allelochemical response by root-secreted chemical signals, three open questions must be addressed: (1) Is plant neighbor detection and allelochemical response a general phenomenon or is it restricted to the specific identity of neighbors? (2) How do researchers demonstrate that the chemical-mediated responses happen belowground rather than in aboveground signaling interactions? (3) Which signaling chemicals are responsible for neighbor detection and allelochemical response in allelopathic plant–plant interactions? It appears from an allelopathy-based wheat–neighbor system that plant neighbor detection and allelochemical response are a general phenomenon. When wheat was paired with 100 other plant species, wheat was able to detect the presence of neighbors in a density-dependent response to neighboring plants regardless of species. Further, in a segregation-based approach with and without root contact, mycorrhizal fungi and soil clearly distinguished aboveground and belowground signaling interactions in neighbor detection and allelochemical DIMBOA response, and the study showed that root exudates are sufficient to induce the response (Figure 1). Importantly, root-secreted (–)-loliolide and jasmonic acid are responsible for the plant neighbor detection and allelochemical response [23]. In particular, root-secreted (–)-loliolide as a general soil-borne signal is common to competitors, and belowground exposure to (–)-loliolide provides an effective signal-mediating response for plants against competitors, and ultimately increases plant survival and fitness.

Although there are likely to be additional signaling mechanisms that could allow species-specific responses, plants are able to commonly detect and respond to their competitors. In cropping systems, the signaling chemicals enable crucial staple food crop plants to detect their competing weeds and respond accordingly to ensure their survival [23,64,130,138]. Plant competitor detection and allelochemical elicitation play important roles in regulating crop–weed inter-specific interactions. Once a cultivated field is infested with weeds, allelopathic crop plants may detect and then enhance the release of allelochemicals to inhibit the weeds, reducing reliance on traditional herbicides for effective weed management. 

Plants have evolved chemical defense mechanisms that produce and release specialized metabolites against interacting competitors. These defense mechanisms start from the recognition of intricate signaling networks while plant defensive metabolites reduce the performance of competing plants to strongly affect plant survival and fitness [19,134,142]. Although aboveground plant–plant signaling interactions and chemical defenses have substantial impacts on plant survival and fitness, relatively less is known about the signaling-mediated belowground interactions. Signals regulating underground plant–plant interactions are whispers in the dark. A key but still poorly understood mechanism for plant neighbor detection and identity recognition is through the perception and recognition of signals derived from root exudates [92].

### 3.3. Relatedness-Mediated Neighbor Recognition

Plant neighbor detection and identity recognition occur among one or more species and within a species, even individuals at a family level. Accordingly, the concept of identity recognition includes species recognition, self/non-self-recognition, kin recognition, and cultivar recognition. Species recognition, that plants can distinguish between members of the same species and those of different species, is described as above in Section 3.2. The self/non-self-recognition arises from the neighbor detection generated by the plant itself. i.e., a plant may also specifically respond to the presence of a competing neighbor by competing or not competing itself [50,143]. Therefore, self/non-self-recognition is identity recognition that allows plants to avoid within-plant competition for resources. The most of research on self/non-self-recognition has particularly focused on the root systems where apparent competition or non-competition between plants can be found [143,144]. For example, the clone plant *Buchloe dactyloides* responds to both self and non-self root systems. When exposed to its own root system, the roots become smaller and shorter. However, coexistence with a non-self root system significantly enhances root growth [144]. Similar root responses to self and non-self phenomena have also been observed in *Ambrosia dumosa* [145], *Glycine max* [146], *Phaseolus vulgaris* var. Kenya [147], *Pisum sativum* [148], and *Trifolium repens* [149]. 

Despite a variety of observations that have been obtained for self/non-recognition via root systems in a relatively short burst of activity, these studies have been criticized for their experimental design because these experiments simply show root growth differently from one larger twin plant. In particular, there is no convincing mechanism that could account for self/non-self-recognition [50]. Due to the flaw in experimental design and the problem of lacking a convincing mechanism, work on self/non-self-recognition has no longer been conducted in the last decade. In contrast, identity recognition has been focused on relatedness-mediated neighbor recognition.

Relatedness-mediated neighbor interactions involve kin recognition, kin discrimination, and kin selection. Kin recognition is the ability to detect the difference in level of relatedness between oneself and another individual, while kin discrimination is a relatedness-dependent cooperative behavior. Plants can distinguish their conspecific neighbors based on their genetic relatedness (i.e., kin recognition) and adjust their ‘behavior’ accordingly (i.e., kin discrimination) [150]. Such kin recognition and kin discrimination have been observed in individuals [151], populations, biotypes [122,152], and cultivar levels [87]. Kin recognition and kin discrimination allow behaviors toward kin groups. Cooperative behavior can be promoted through kin selection. Kin selection theory states that an altruistic trait can be favored by natural selection when the altruist and the recipient are related [153]. Importantly, kin recognition is associated with kin discrimination, but kin recognition and associated kin discrimination are not necessary for kin selection to occur [6].

Kin recognition is identity recognition at the level of siblings vs. non-siblings from the same population and species. Kin recognition in plants was proposed in 1980 [154] and first found in an annual plant, *Cakile edentula*, in 2007. The annual plant greatly expanded its root systems in the presence of unrelated plants but reduced root growth and competition with relatives, allowing greater shared access to resources [151]. Subsequent studies have shown that many plants are able to differentiate relatives from unrelated plants from the same population. Such kin recognition occurs in various types of plant species, ranging either from gymnosperms to angiosperms, or from wild species to crop plants [87,122,152,155,156]. The ability to detect relatedness would allow plants to discriminate closely from distantly related neighbors and optimize response strategies for the composition of their local neighborhood [6,150,157,158]. Plant kin recognition, without compromising individual fitness, enhances the survival chances of population offspring, playing a positive role in plant population reproduction and community assembly.

Cooperation arising from kin recognition makes for behaviors toward relatives that promote the increase of related offspring. Reducing the energy devoted to competitive organs allows for greater allocation to reproduction in kin groups [159,160,161]. Kin recognition and kin discrimination in crop plants can result in less intra-specific competition, maximizing stand performance and increasing yield [87,162]. For example, rice cultivars with the ability for kin recognition are capable of distinguishing between closely and distantly related cultivars and respond to them by altering biomass allocation, increasing grain yield in closely related cultivar mixtures [87]. Therefore, kin recognition and kin discrimination in crop plants may be a potential mechanism responsible for performance and yield in cultivar mixtures [5,87,151]. Different from naturally occurring species where kin represents plants sharing the same mother as either full or half siblings, artificial selection generates crop cultivars that are genetically and morphologically uniform. In the case of highly self-pollinating cultivars, the coefficient of relatedness is very close to 1, i.e., r ≈ 1 [6,156,163]. Therefore, kinship in self-pollinated crop plants is functionally represented by closely related cultivars rather than siblings within a natural species, and kin recognition in crop plants occurs at the cultivar level [87,142]. 

Kin recognition and kin discrimination involve signaling interactions among intra-specific neighbors [150,161,164]. Most evidence suggests that kin recognition is mediated by a chemical mechanism and requires active secretion by roots, as recognition can take place under root segregation or before physical contact occurs [87,164]. A few studies have shown that root exudates can invoke kin and non-kin responses. However, it is not clear which root-secreted signaling chemicals are responsible for these responses [87,128,165]. A specific root-secreted allantoin has a role in kin recognition in rice lines [87]. However, the nitrogen-rich allantoin may not be the signal of relatedness but rather the effect of the underlying signal [84]. Root exudates contain signaling chemicals, such as (–)-loliolide, a common signal for neighbor detection [23,132,133]. This plant–plant signaling is reminiscent of the role of (–)-loliolide in relatedness-mediated neighbor identity recognition and discrimination. (–)-Loliolide may function as a biochemical mechanism to discriminate closely related cultivars from distantly related cultivars (that have therefore not been published).

Ecological and evolutionary approaches to improving crop mixtures have been commonly used throughout the world [2,166]. The benefits of mixed cropping systems have been attributed to niche complementarity, facilitation, and within-species diversification in inter-specific interactions [166,167,168] but have rarely been invoked in relatedness-mediated intra-specific interactions. Kin recognition and kin discrimination among crop plants are predicted to increase yield by reducing intra-specific competition and shifting resource allocation to reproduction [87,150,169]. Preferentially reducing competitive effects on relatives may improve our understanding of the processes at play in kin recognition to develop cultivar mixtures that increase grain yields in the limited area suitable for agriculture.

## 4. Chemically Mediated Root–Soil Interactions and Plant–Soil Feedback

### 4.1. Root–Root Interactions and Root Placement Patterns

Roots function primarily in the acquisition of nutrients and water and interacting with soil. Therefore, root systems have recognizable developmental plans that are primarily driven by resource availability. However, root–root interactions are much more sophisticated than previously thought as regards foraging for resources only. Plant roots detect and respond to complex physical and chemical cues from the soil environment [170,171]. The ability of roots to avoid obstacles has been observed for over a century. The phenomenon and its mechanisms may be explained by chemically mediated root detection and action. The roots of *Pisum sativum* can detect and avoid growth towards obstacles. Importantly, *Pisum sativum* root-secreted allelochemicals to inhibit their own roots, resulting in root navigation by self-inhibition [172]. Such obstacle avoidance by self-inhibition could optimize root systems, increasing plant performance by limiting resource allocation. Soil compaction limits the ability of roots to penetrate harder soils, reducing root growth. A recent study has shown that plant roots sense soil compaction through restricted ethylene diffusion. In a compacted soil environment, ethylene accumulated in root tissues acts as a warning signal for roots to restrict root growth towards compacted soils [118]. Importantly, roots can chemically identify and respond to roots of conspecific and inter-specific neighbors and even self from non-self roots and kin from non-kin roots within a family [40,87,143,144,151], resulting in a series of root–root interactions that determine belowground ecological processes to a large extent. Any change in root–root interactions is likely to alter the soil resource availability, and subsequently, the plant fitness.

In the interactions among roots of *Ambrosia dumosa* and *Larrea tridentata* in the Mojave Desert community, Larrea roots released allelochemicals into the soil to inhibit either Larrea or Ambrosia roots in their vicinity. However, Ambrosia roots inhibited conspecific contacted roots only, indicating their different root–root interactions are mediated by allelopathy rather than simple competition for a limiting resource. [145]. Chemically mediated root–root interactions have been extensively observed in crop–weed interactions and mixed-species plantations. The paddy weeds detect and respond to the presence of rice by altering root growth and distribution in a cultivar-dependent manner. In the presence of common rice cultivars, the root growth and distribution of paddy weeds are intrusive, unresponsive, and avoidant towards rice roots. However, the roots of paddy weeds avoid the roots of allelopathic rice cultivars [60]. In the *M. macclurei* and Chinese fir mixed-species plantation, Chinese fir root systems develop differently in the presence of their own roots than *M. macclurei* roots. In particular, Chinese fir root growth increased towards neighboring *M. macclurei* roots. This response was attenuated by activated carbon, indicating that there were chemically mediated root–root interactions in the mixed-species plantation [59]. 

Roots may discriminate the roots of self/non-self and respond by either stimulating the growth of self-roots or inhibiting the intrusion of non-self-roots. Self/non-self-root–root recognition is carried out with electrical or turgor-associated signals [91,173], such as the electrical signal-mediated root communication between *A. dumosa* and *L. tridentat* [157], and root self-recognition by differential internal oscillatory signals in *Pisum sativum* and *Buchloe dactyloides* [143,144]. Nonetheless, self- and non-self-root–root interactions are likely to be mediated by root-secreted chemical signals alone or in combination with physical signaling. 

Roots can detect and discern roots from close relatives and unrelated plants from the same species or family through the root exudates [165]. Rice cultivars with the ability for kin recognition can detect the presence of closely and distantly related cultivars, and respond to them by adjusting their root placements and biomass. A root-secreted allantoin may be responsible for the relatedness-mediated root–root interactions [87]. Furthermore, the intra-specific kin recognition contributes to inter-specific allelopathy in allelopathic rice interference with paddy weeds. Closely related cultivar mixtures not only reduce the production of rice allelochemicals by lowering the defense cost, but also are capable of preferential root placement towards weeds [142].

In response to neighbor roots and root–root interactions, plants actively alter root placement patterns to buffer root competition and provide potential advantages for their own growth and fitness. Generally, root–root interactions represent intrusive (approaching, over-proliferation), avoidant (repelling, underproliferation), or unresponsive patterns [60,174]. However, root placement responses are reciprocal with neighboring roots. Therefore, pairwise plant–plant interactions can potentially generate nine combined patterns. A recent study has experimentally documented the occurrence of these nine root placement patterns, including intrusive-intrusive, intrusive-avoidance, intrusive-unresponsive, avoidance-intrusive, avoidance-avoidance, avoidance-unresponsive, unresponsive-intrusive, unresponsive-avoidance, and unresponsive-unresponsive patterns, in pairwise allelopathic wheat and 60 other plant species systems [63]. Importantly, the neighbor-modulated root placement patterns are mediated by both allelochemicals and signaling chemicals. Signaling (–)-loliolide from neighbor species triggered wheat allelochemicals to inhibit root growth with degrading starch grains and inducing an uneven distribution of auxin in neighbor species roots, altering root placement patterns [63].

Neighbor-modulated root–root interactions and root placement patterns driven by root-secreted allelochemicals and signaling chemicals are important mediators of belowground interactions, impacting plant fitness, reproduction, and survival [84,128,173]. Root placement patterns alter the spatial and temporal distribution of root systems in the soil, whereas the spatial and temporal distribution of root systems affect the dormant microbes of over 95% of the microbial biomass in soil. These dormant soil microbes can be activated by exudates from arriving roots. In turn, activated microbes may induce root exudation [175]. Accordingly, root placement patterns may determine the rhizosphere microbiome and soil microbial community assembly, ultimately altering root–soil interactions and belowground ecological processes.

### 4.2. Soil Mobility and Microbial Interactions

Plant–plant chemical interactions are mediated by allelochemicals and signaling chemicals, either volatiles or root exudates from plants into the environment, mostly into the soil. The action of allelochemicals and signaling chemicals in soil requires their presence in the vicinity of the target plants. Thus, allelochemicals and signaling chemicals endure under soil processes, such as retention, transport, and transformation, once released [176]. Of these processes, the mobility of allelochemicals and signaling chemicals, as well as their bio-transformed by soil microbes, are a determinant for belowground plant–plant interactions. The mobility and microbial degradation affect the final destination and concentration of the allelochemicals and signaling chemicals in the soil surrounding neighbors, and subsequently, their activities.

The mobility of 10 typical allelochemicals, including ferulic, p-hydroxymandelic, phydroxybenzoic and vanillic acids, vanillin, coumarin, daidzein, 1α-angeloyloxycarotol, DIMBOA, and m-tyrosine, was evaluated by using a soil thin-layer chromatography (soil TLC) combined with a bioassay approach [177]. There were significant differences in mobility factor (*Rf*) among these allelochemicals. Four phenolic acids (ferulic, p-hydroxymandelic, phydroxybenzoic, and vanillic acids) had very poor mobility (*Rf* < 0.1). In contrast to phenolic acids, phenolic aldehyde and lactone (vanillin and coumarin) showed excellent mobility (*Rf* > 0.5) in soil. Whereas daidzein (flavonoids), 1α-angeloyloxycarotol (terpenoids), DIMBOA, and m-tyrosine (non-protein amino acid) showed good mobility and their *Rf* values range from 0.24 to 0.32. Accordingly, the mobility factor is dependent on the structural specificity of allelochemicals, as polar allelochemicals could not easily be moved from the allelopathic plants to target species in the soil environment. However, binary mixtures of these allelochemicals led to an increase in mobility factors for selected combinations [177]. Similarly, the mobility of the four signaling chemicals, jasmonic acid (*Rf*, 0.21), salicylic acid (*Rf*, 0.07), (–)-loliolide (*Rf*, 0.68), and luteolin (*Rf*, 0.32), in soil was evaluated by using a soil TLC. Among them, the root-secreted signaling chemical (–)-loliolide had a higher soil mobility and could easily be moved from the rhizosphere to bulk soil [23]. Therefore, (–)-loliolide may be a soil-borne signaling chemical in plant–plant signaling interactions.

Plants release allelochemicals and signaling chemicals at rates of significance to interact with soil microbes. Allelochemicals and signaling chemicals are able to exert an effect on soil microbes. In turn, soil microbes consume and decompose allelochemicals and signaling chemicals. Allelopathic rice seedlings released 5,4′-dihydroxy-3′,5′-dimethoxy-7-*O*-β-glucopyranosylflavone into soil, where the glucopyranosylflavone rapidly hydrolyzed glucose by soil bacteria into allelochemical 5,7,4′-trihydroxy-3′,5′-dimethoxyflavone [178]. The allelochemical not only inhibited the root growth of paddy weeds but also reduced the culturable microbial population and total microbes in the soil. Therefore, allelopathic rice can modify the soil microbial community through root-secreted allelochemicals and their microbe-transformed production [179]. Similar results occur in wheat and barley allelopathy. Allelopathic wheat root-secreted DIMBOA and then microbe-transformed into 6-methoxy-benzoxazolin-2-one (MBOA) as allelochemicals against pests. There was a positive relationship between wheat DIMBOA and MBOA levels and soil microbial population. DIMBOA associated with yielding MBOA increased soil fungi, affecting the soil microbial community structure [140]. Barley produces the allelopathic alkaloids gramine and hordenine, whose biosynthesis and accumulation are preferentially located in roots [180,181]. The allelopathic alkaloids impact the community structure of field soil bacteria. Gramine caused the proliferation of many potentially beneficial strains. Furthermore, gramine biosynthesis was accompanied with the association of distinct bacterial communities in the soil [181].

Soil disease caused by a continuous monoculture of crop plants and woody trees was involved in the build-up of soil pathogens. Allelochemicals may contribute to the shift in soil microbial communities in continuously cultivated fields and plantations. The levels of allelochemicals in the rhizosphere correlate with the presence of various microbes in soil [140,182,183]. A case study indicated rhizosphere isoflavone (daidzein and genistein) levels and their relation to soil microbial community structure in a 13-year experiment of continuous soybean monocultures. As a result, soil microbial community structures were induced by both daidzein and genistein, and thereby daidzein and genistein in the soybean rhizosphere may act as allelochemicals in the interactions between the root and soil microbial community in a long-term mono-cropped soybean field [183]. Similar results have been found in 25-year-old monospecific Chinese fir plantations. There were negative relationships among allelochemical cyclic dipeptide concentration, microbial community composition, and Chinese fir root biomass in plantations. In particular, allelochemical cyclic dipeptide accumulated potentially pathogenic fungi, altering the soil microbial compositions and community structures. Allelochemical-mediated soil microbial community with a negative feedback result in a productivity decline of continuous Chinese fir monocultures [76,77]. Further clarification of the root–microbe interactions mediated by allelochemicals may contribute to solving the problem of continuous monocultures in cropping systems and tree plantations.

Extensive research has shown that soil microorganisms are an important determinant of allelopathic activity [182,184,185]. However, determining how allelochemicals affect the soil microbiome and how soil microbial changes mediate plant performance is challenging. Recent microbiome technology development makes an effort directed toward understanding the impact on soil microbial composition and the community structure of allelochemicals in the soil environment. Several studies have shown allelochemical-selected microbiomes and their effects on plant performance [186,187]. Allelochemicals affect soil microbiome structure by altering core bacterial and fungal taxa. The allelochemical-selected soil microbiome mediated plant performance across 13 plant species, mitigating allelopathic inhibition of plant productivity [186]. Rhizosphere microbial communities are vital for plant performance. A recent study indicates metagenomic insights into the responses of rhizobacteria and their alleviation role in licorice allelopathy. Allelochemical glycyrrhizin inhibited the growth and development of licorice, resulting in autotoxicity. However, specific rhizobacteria, such as the *Novo-sphingobium* genus, accounted for a relatively high proportion of the enriched taxa and appeared in metagenomic assembly genomes, such as degraded glycyrrhizin, to alleviate the allelopathic autotoxicity [187]. The results provide potential implications for resolving the problem of continuous monocultures using an allelochemical-selected rhizobacterial community.

Despite increasing knowledge of the allelochemicals involved in the soil microbial community, little is known about signaling chemicals in the rhizosphere and interactions with the soil microbial community [188]. The nitrogen-rich allantoin, as a potential root-secreted signaling chemical, significantly stimulated populations of bacteria and actinomycetes but had no effect on fungi in paddy soil [189]. Signaling allantoin increased the ratio of anaerobe:aerobe and bacteria:fungi but decreased the ratio of Gram-negative bacteria:Gram-positive bacteria, and the fungal signature lipid marker 18:2ω6,9c under flooded rice soil. The allantoin-induced shifts in microbial community composition increased microbial diversity as well as the living microbial biomass in rice soil [190]. 

Several studies have shown the role of root-secreted signaling (–)-loliolide in soil microbial compositions and community structures [116,117,124]. (–)-Loliolide affected the signature lipid biomarkers of soil bacteria, actinobacteria, and fungi, resulting in changes in soil microbial community structures [191]. In penoxsulam-resistant barnyardgrass-mediated root microbial communities, (–)-loliolide and jasmonic acid in the root exudates were correlated with the core microbes in the rhizosphere soil [192]. Similar correlations between (–)-loliolide and rhizosphere bacteria occurred in Arabidopsis, indicating that Arabidopsis may regulate the rhizosphere bacteria through root signaling (–)-loliolide [124]. In addition, ethylene is a typical air-borne chemical signal to participate in plant neighbor detection [116] and to induce a shade avoidance response [117]. A recent study has shown that ethylene emitted from peanut roots can reorganize the rhizosphere microbial network, and the reassembled rhizosphere microbial community can provide more available nutrients for peanut roots to promote growth and reproduction [193].

### 4.3. Belowground and Aboveground Interactions

**Figure 2:**
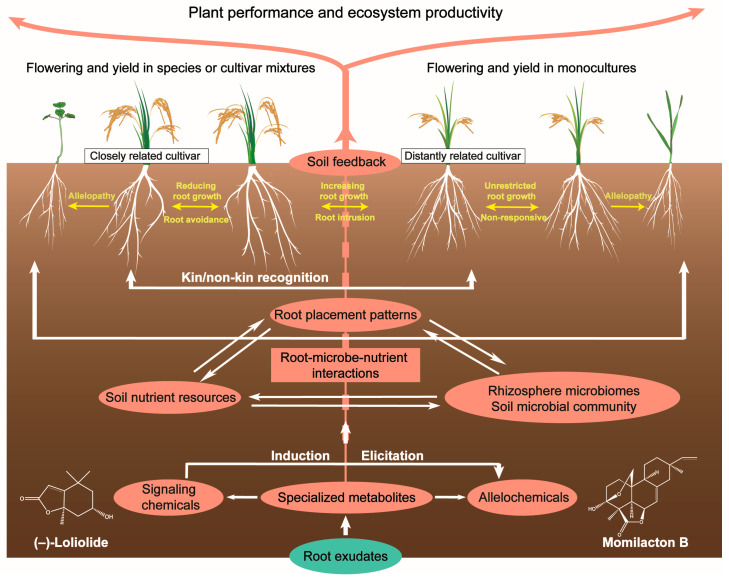
Chemically mediated root–soil interactions and plant–soil feedback.

Soil microbes play a crucial role in belowground ecological processes, such as nutrient cycling and antibiotic resistance [193,194,195,196]. Importantly, soil microbial communities affect aboveground performance, either through inter-specific or conspecific plants, or even kin and non-kin individuals [116,142,197]. Plants can shape soil microbiota through root-secreted allelochemicals and signaling chemicals [140,175,179,184,198]. In turn, chemically mediated soil microbial communities drive plant–soil feedback, affecting flowering and reproduction [22,199,200], and thus altering plant fitness (Figure 2).

Flowering is a key means for plant species to obtain maximum reproductive capacity. The flowering of plants is mostly aboveground leaves sensing environmental factors. Recent studies have shown that rhizosphere and soil microbial communities can alter the selection intensity of plant flowering phenology and flowering time [199,201,202,203]. In particular, root-secreted signaling chemicals can recruit specific microbes to alter the rhizosphere and soil microbial communities, impacting the flowering of plants [124,201]. A novel metabolic network in which soil microbiota influenced plant flowering time has been found in Arabidopsis, where the root exudates affected plant rhizosphere microbiota, modulating flowering time by IAA production [201]. Furthermore, the flowering time of Arabidopsis shifted with root signaling (–)-loliolide and rhizosphere bacteria. There were differential rhizosphere and soil bacterial communities between two genotypes with and without root-secreted (–)-loliolide, suggesting that the flowering time of Arabidopsis may be mediated by rhizosphere bacteria with root-secreted chemical signals [124]. Despite microbiome being a major contributor to phenotypic variation, flowering time may be determined by specific rhizobacteria rather than the whole microbiome. A recent study has shown that rhizosphere bacteria (*Cupriavidus metallidurans*, *Bacillus*, *Solibacillus*, and *Planococcus*) affect the flowering time of Arabidopsis [204]. 

Chemically mediated belowground ecological interactions among roots, microbes, and nutrients can feed back to the plant aboveground, affecting growth, flowering, and reproduction [200,201,202,203]. Plants release allelochemicals and signaling chemicals to alter the rhizosphere and soil microorganisms that increase and decrease nutrient bioavailability, resulting in a positive or negative plant–soil feedback (Figure 2). Plant–soil feedback has been recognized as an important mechanism behind intra- and inter-specific interactions, affecting plant performance, species diversity, and community structure [205]. Allelochemicals and signaling chemicals affect plant inputs into the soil subsystem via litter and rhizodeposits. In particular, root exudate metabolites drive plant–soil feedback on growth and defense by shaping the rhizosphere microbiota and modulating microbial succession [200,206,207,208]. These chemically mediated interactions may cause specific plant–soil feedback where the match between the species identity of living roots plant nutrition is evaluated [200,205].

The intricate chemical communication plays an instrumental role in regulating the balance and function of the plant–microbe–soil system. The communication network profoundly affects the growth, metabolism, and interactions of microorganisms, thereby influencing the structure and function of soil ecosystems. Different types of allelochemicals and signaling chemicals exhibit selective effects on different microorganisms, thereby governing the composition and abundance of microbial communities. The regulation of such interactions is a complex process that involves the integrated effects of multiple factors and signaling pathways. Moreover, the specific mechanisms and effects may vary in different plant species and under different environmental conditions, necessitating further research and exploration.

## 5. Biosynthesis and Molecular Mechanisms of Allelochemicals and Signaling Chemicals

### 5.1. Biosynthesis and Mechanisms of Allelochemicals

Plants biosynthesize a wide variety of allelochemicals including phenolics (simple phenolic acids, flavonoids, coumarins, and quinones), terpenoids (monoterpenes, sesquiterpenes, diterpenes, and steroids), nitrogen-containing chemicals (alkaloids, benzoxazinoids, and cyanogenic glycosides), and many other chemical families [18,19]. These allelochemicals are regulated by various genes, including those that encode transcription factors, subsequently determining allelopathic potentials [209]. 

The biosynthesis of phenolic allelochemicals is related to the shikimic acid and the phenylpropanoid pathways. In the shikimic acid pathway, hosphoenolpyruvate synthetase (PEPs) and erythrose-4-phosphate (E4P) are catalyzed into phenylalanine under the 3-deoxy-d-arabino-heptulosonate-7-phosphate synthase (DAHPS), 3-dehydroquinate synthase (DHQS), and shikimate dehydrogenases (SDH) [210]. The phenylpropanoid metabolism pathway begins with phenylalanine; under the catalysis of phenylalanine ammonia-lyase (PAL) and cinnamic acid 4-hydroxylase (C4H), it transforms into p-coumaric acid. Through the catalysis of enzymes, such as p-coumarate-3-hydroxylase (C3H), caffeate 3-O-methyltransferase (COMT), ferulate-5-hydroxylase (F5H), and 4-coumaroyl-CoA ligase (4CL), p-coumaric acid is further converted into caffeic acid, ferulic acid, sinapic acid, p-coumaroyl-CoA, and chlorogenic acid [210]. Finally, under the catalysis of chalcone synthase (CHS) and chalcone isomerase (CHI), flavanone 3-hydroxylase (F3H), flavanone 3′-hydroxylase (F3′H), flavonol synthase (FLS), and flavone synthase (FNS), p-coumaroyl-CoA is transformed into flavones, flavonols, and other related flavonoids [210,211]. In addition to key enzymes before, TFs such as MYB, bHLH, WD-WDR, NAC, and WRKY can coordinately regulate the expression of enzymatic genes at the transcriptional level in a spatial manner [212]. 

Phenolic acids are common allelochemicals and their molecular mechanisms have been revealed. In maize, the constitutive expression of ZmMyb-IF35 can promote ferulic acid and chlorogenic acid accumulation [213]. The specific expression of *StAN1* and *StMTF1* in *Solanum lycopersicum* promotes the synthesis of chlorogenic acid and caffeic acid [214]. The constitutive expression of *AtMYB12* in *Arabidopsis thaliana* increases the content of chlorogenic acid [215]. *SmMYB98* positively regulates the synthesis of salvianolic acids in *Salvia miltiorrhiza*, and *SmMYB2* significantly increases the level of salvianolic acids by upregulating the synthesis gene *CYP98A14* [216]. In rice, the transcriptional activator VP64 can upregulate *OsMYB57*, inducing the synthesis of phenolic acids and enhancing rice allelopathy against barnyardgrass [209]. 

Flavonoids are a crucial component of phenolic allelochemicals. In Arabidopsis, *AtMYB11*, *AtMYB12*, and *AtMYB111* can independently activate genes encoding CHS, CHI, F3H, and FLS, and then determine the content of flavonols, collectively [217]. *AtMYB4* has the ability to inhibit the expression of *AtMYB7*, and silencing *AtMYB4* can increase the biosynthesis of flavonols in *Arabidopsis thaliana* [218]. *MYBP3* and *MYBP4* can enhance the flavonol level by activating related genes in both *Arabidopsis thaliana* and *Gentiana trifloral* [219]. Furthermore, the transcription factor MYB regulates flavonol synthesis in *Vitis vinifera*, *Malus pumila*, *Fragaria ananassa*, *Glycine max*, and *Ginkgo biloba* [214,220,221,222]. Transient expression of *GmMYBJ3* can activate CHS8 and CHI1A, promoting the synthesis of isoflavones in soybeans [223]. Moreover, in the roots of leguminous plant *Callerya speciosa*, nine CsMYBs TFs are identified to form a regulatory network with bHLH, increasing isoflavone content by activating synthesis genes [224]. Overexpression of *CaMYB39* activates early flavonoid biosynthetic genes (EBG), including *SYNTHASE2*, which can induce the biosynthesis of flavanones, flavonols, isoflavones, and others [225].

Quinone is another important group of phenolic allelochemicals, including benzoquinones, naphthoquinones, anthraquinones, and others. Juglone is a classic naphthoquinone allelochemical derived from several pathways, including the o-succinylbenzoate pathway (OSB), the 4-hydroxybenzoic acid (4HBA)/geranyl diphosphate (GPP) pathway, the acetate-polymalonate pathway, and the homogentisate (HGA)/mevalonic acid (MVA) pathway [226]. Recent studies indicate that specialized 1,4-naphthoquinones may evolve from 1,4-dihydroxynaphthoic acid (DHNA), an intermediate in the biosynthesis of phylloquinone [227]. In particular, the naphthalenoid moiety of juglone in *Juglans nigra* originates from DHNA produced via the phylloquinone pathway [228].

Terpenoids constitute the second-largest group of allelochemicals, characterized by poor water solubility, making it challenging for rainwater to leach them into the soil [229]. The precursors of terpenoids are synthesized through two pathways: the MVA pathway that occurs in the cytoplasm and the methylerythritol phosphate (MEP) pathway that takes place in the plastids. The MVA synthesis pathway involves six enzyme-catalyzed reactions, starting from three molecules of acetyl CoA and being catalyzed by enzymes such as AACT, HMGS, HMG CoA, and others to produce isopentenyl diphosphate (IPP). The MEP synthesis pathway is completed through seven enzyme-catalyzed reactions, beginning with the condensation of D-glyceraldehyde-3-phosphate and pyruvate. Enzymes such as DXS, DXR, CMS, and HDR catalyze the formation of IPP and dimethylallyl pyrophosphate (DMAPP). Finally, under the catalysis of different enzymes, IPP and DMAPP go through various condensation reactions to produce terpenoids [230]. 

The synthesis genes for various terpenoids have been identified. In tea trees, genes such as *CsTPS*, *CsFPS*, *CsOCS2*, and *CsBOS1* have been identified to regulate the levels of allelochemicals in tea plants, such as (E)-nerolidol, (E)-β-ocimene, geraniol, β-myrcene, D-limonene, and (E)-β-farnesene [231,232]. A-type diterpenoid allelochemical momilactone is catalyzed by class II diterpenoid cyclases and class I diterpenoid synthetases, respectively. In rice, *CYP76M8* is responsible for the generation of momilactone [233]. In addition, TFs involved in the synthesis of plant terpenoids include AP2/ERF, bHLH, and MYB. These TFs can effectively regulate multiple enzyme genes in the terpenoid biosynthetic pathway. Therefore, achieving global regulation of the terpenoid biosynthetic pathway through transcriptional regulatory networks is more effective.

Compared to phenolics and terpenoids, nitrogen-containing allelochemicals are relatively scarce, mainly comprising alkaloids, such as gramine and hordenine [180,181] and non-protein amino acids, such as δ-hydroxynorleucine, L-canavanine, L-DOPA, L-meta-tyrosine, β-(3-isoxazolin-5-one-2-yl)-alanine, and mimosine [18,19]. Solanidine or solanine is a general term for glycosylated derivatives of steroidal alkaloids (SAs) in plants. Among them, α-chaconine and α-solanine are the most abundant, constituting 90% of the total solanidine content [234]. Chemically, solanidine is a glycosylated derivative of cholesterol and its synthesis involves a multi-gene process divided into three stages: (1) acetyl-CoA is converted into terpenoid compounds; (2) synthesis of cycloartenol; and (3) reduction of cycloartenol to cycloartanol and further synthesis into cholesterol, which is then transformed into solanidine. Finally, solanidine is glycosylated into α-solanine and α-chaconine through three different solanidine glycosyltransferases (SGT) [234]. 

In addition to alkaloids and non-protein amino acids, there are other nitrogen-containing allelochemicals, with the most representative being benzoxazinoids (BXs) produced by wheat and maize. Natural BXs are divided into three groups based on their N-substituents: lactams, methyl derivatives, and hydroxamic acids [235]. The BX synthesis pathway is a branch of the tryptophan biosynthetic, initiating with 1-(2-carboxyphenylamino)-l-deoxyribulose-5-phosphate that catalyzes by indole-3-glycerolphosphate synthase (IGPS) to form indole-3-glycerol phosphate, followed by the action of indole-glycerol phosphate aldolase (Bx1) and four consecutive cytochrome P450 enzymes (Bx2–Bx5) and glucosyltransferases (Bx8/Bx9) to generate DIBOA-Glc (2,4-dihydroxy-1,4-benzoxazin-3-one-glucoside) [56,236]. DIBOA-Glc is further transformed by O-methyltransferases (BX10 to BX12) into 2-hydroxy-4,7-dimethoxy-1,4-benzoxazin-3-one glucoside (HDMBOA-Glc). When DIMBOA-Glc and HDMBOA-Glc stored in vacuoles come in contact with specific glucosidases, they are converted into DIMBOA and MBOA, respectively [237].

Although the genes involved in the biosynthesis of BXs have been extensively investigated, the transcriptional regulation of BX genes is beginning. Some transcription factors have been reported to play a role in maize. Upon biotic stress, *ZmWRKY75*, *ZmMYB61*, *ZmNAC35*, and *ZmGRAS37* are upregulated in maize, leading to the upregulation of *ZmBx1* and *ZmBx13* expression and an increase in BX level [238]. Zhou et al. (2020) identified transcription factor families, such as MYB, NAC, and GRAS, through gene regulatory networks (GRNs) that can regulate BX genes in maize [239]. However, these studies were conducted using maize transcriptomics methods and did not directly test the functions of identified transcription factors. The following studies reported that in wheat, *TaMYB31* can bind to the promoters of Bx1 and Bx4, catalyzing the biosynthesis of BXs in response to biotic and abiotic stress [240].

### 5.2. Biosynthesis and Mechanisms of Signaling Chemicals

Plants can rely on information mediated by signaling chemicals to recognize neighbors and adjust their survival strategies. Volatile organic compounds (VOCs) emitted from plants are vital signaling chemicals, including green leaf volatiles (GLVs), terpenes, methyl jasmonate (MeJA), methyl salicylate (MeSA), ethylene, and indole. Root exudates also are key components of plant–plant signaling interactions but the root-secreted signaling chemicals have not been well-known. So far, strigolactones (SLs), (–)-loliolide, jasmonic acid (JA), and allantoin have been identified as root-secreted signaling chemicals that are responsible for belowground signaling interactions (Table 1). Once these signaling chemicals are received and recognized by neighboring plants, they may trigger a series of plant responses through signal transduction, changes in the transcriptome, proteome, and metabolome, resulting in corresponding functional alterations [84,89]. Therefore, the signaling chemicals and their biosynthetic pathways as well as regulatory mechanisms have been investigated in the past decades. 

Terpenes and terpenoids are major signaling chemicals in plants. Abscisic acid, brassinosteroids, cytokinins, gibberellins, strigolactones, and (–)-loliolides are all classified as terpenoids. The biosynthesis of terpenoids involves three stages: (1) The generation of C5 precursors isopentenyl pyrophosphate (IPP) and its isomer dimethylallyl pyrophosphate (DMAPP); (2) The generation of direct precursors farnesyl diphosphate (FPP), geranyl diphosphate (GPP), geranylgeranyl diphosphate (GGPP), etc.); (3) The terpenoid formation and modification stage [241]. The synthesis of IPP and DMAPP occurs through either the mevalonic acid (MVA) pathway, which is located in the cytoplasm and utilizes acetyl-CoA as a substrate, or the deoxyxylulose-5-phosphate (DXP) or methylerythritol phosphate (MEP) pathway, which is located in the plastids and utilizes pyruvic acid and glyceraldehyde-3-phosphate as substrates [242]. The MVA pathway provides precursors (such as FPP, C15) for the biosynthesis of sesquiterpenes, triterpenes, and sterols (such as brassinosteroids). The rate-limiting enzyme in this pathway is 3-hydroxy-3-methylglutaryl-coenzyme A reductase (HMGR). Meanwhile, the MEP pathway provides precursors for the synthesis of monoterpenes (GPP, C10) and precursors for the synthesis of diterpenes, gibberellins, and carotenoids (GGPP, C20). The key enzyme in this pathway is 1-deoxyxylulose-5-phosphate synthase (DXPS). Finally, the direct precursors are catalyzed by terpene synthases to produce various terpenes [243].

The biosynthesis of terpenoids is regulated by various transcription factors. Artemisinin is a sesquiterpene lactone isolated from *Artemisia annua*, and several transcription factors associated with artemisinin biosynthesis have been identified. The sesquiterpene synthase amorpha-4,11-diene synthase (ADS) and P450 monooxygenase (CYP71AV1) are two key enzymes involved in artemisinin biosynthesis. Two JA-sensitive AP2/ERF transcription factors, AaERF1 and AaERF2, induce the promoter activity and promote the transcription of ADS and CYP71AV1. Overexpression of *AaERF1* or *AaERF2* leads to increased accumulation of artemisinin and artemisinic acid [244]. The AabHLH1 protein could bind to E-box cis-elements in the promoters of ADS and CYP71AV1 and had transcription activity in yeast. In addition, transient expression of AabHLH1 in *A. annua* leaves increased the transcription levels of genes involved in artemisinin biosynthesis (such as ADS, CYP71AV1, and HMGR). These results indicate that AabHLH1 positively regulates artemisinin biosynthesis [245]. The synthesis of allelochemical saponins in *Medicago truncatula* and *Conyza blinii* interaction is regulated by bHLH and the jasmonic acid-responsive transcription factor WRKY, respectively. The bHLH transcription factors TSAR1 and TSAR2 impact saponin biosynthesis in *M. truncatula* by regulating the expression of *HMGR1* [246], CbWRKY24 enhances the total saponin content in *C. blinii* by upregulating the expression levels of multiple MVA pathway genes [247].

Generally, volatile terpenoids participate in plant chemical communication by modulating the expression patterns of key genes, activating relevant signal transduction pathways. Monoterpene volatiles regulated the expression of Arabidopsis transcription factors or defense genes, inducing the accumulation of methyl jasmonate [110]. The release of 4,8-dimethylnona-1,3,7-triene (DMNT) from tea plants damaged by *Ectropis obliqua* induced the expression of *LOX1* and *LOX3* in neighboring plants [111]. *Fusarium oxysporum* infection in tomatoes revealed the release of different volatiles from susceptible and resistant tomato plants. In resistant tomato plants, several genes involved in volatile biosynthesis and fatty acid derivative pathways (such as *MTS1*, *TomloxC*, *TomloxD*, *AOS*, and *JAZ7*) are significantly induced by *F. oxysporum*, activating the JA pathway [248]. *Diaphorina citri* is involved in the recognition process of host plants through the regulation of genes encoding odorant-binding proteins (OBPs) and chemosensory proteins (CSPs) such as *DcitOBP3*, *DcitOBP6*, *DcitOBP8*, *DcitOBP9*, *DcitCSP1*, and *DcitCSP12* [249]. Nerolidol, as a volatile signaling chemical, participated in regulating the expression of *MAPK* and *WRKY* genes and proteins, defense-related signaling molecules, and H_2_O_2_ levels in tea plants [112].

Strigolactone (SLs), as a type of carotenoid-derived sesquiterpenoid, serves not only as an internal hormone but also as a signaling chemical mediating plant–root interactions in the rhizosphere [250]. SLs are synthesized through the carotenoid pathway. β-Carotenoid isomerase (D27), carotenoid cleavage dioxygenase 7 (CCD7), carotenoid cleavage dioxygenase 8 (CCD8), and cytochrome P450 monooxygenase (P450) play an important role [251]. Additionally, AtD14, MAX2 encoding an F-box protein, and the transcription factors BRC1/2 are involved in the strigolactone signaling pathway in Arabidopsis [251]. Strigolactones not only participate in stimulating the germination of parasitic plants like striga but also play a crucial role in the symbiosis between plant roots and arbuscular mycorrhizal fungi [252]. Recent studies have found that root-secreted strigolactones promote plant symbiosis with beneficial microorganisms and participate in the interaction between plants and pathogenic microorganisms. For example, SLs play a negative regulatory role in the defense of rice against the rice blast fungus (*pyricaria oryzae*). The reduction in strigolactone levels in rice enhances its defense against the rice blast fungus by activating jasmonic acid and sugar signaling pathways, as well as accumulating flavonoid phytoalexins [252].

(–)-Loliolide, a β-carotene derivative, serves both exogenous and endogenous roles in plant–plant signaling interactions [132,133,137]. (–)-Loliolide are commonly found in the root exudates and participate in plant–plant allelopathic interactions, inducing the production of allelochemicals and upregulating the expression of key genes involved in the biosynthesis of allelochemicals, such as the rice allelochemicals tricin (*CYP75B3* and *CYP75B4*) and momilacton B (*CPS4*, *KSL4*, and *MAS*) [130]. (–)-Loliolide triggered the production of wheat allelochemicals by upregulating the expression of DIMBOA biosynthetic genes, especially *Taglu*, inducing the expression of defense-related genes, and activating defense-related signaling pathways accompanied by an increase in jasmonic acid and H_2_O_2_ concentrations [132]. A recent study has found that (–)-loliolide participates in regulating the defense response and altering the flowering time of tobacco by modulating the expression patterns of tobacco defense and flowering-related genes [137]. 

Salicylic acid (SA) is an important plant defense hormone. The biosynthesis of SA begins with chorismate and proceeds through the isochorismate synthase (ICS) and phenylalanine ammonia-lyase (PAL) pathways. SA is recognized by receptor proteins such as NPR1 and NPR3/4. SA enhances its DNA binding ability by phosphorylating TGA3 and promotes the formation of the NPR1-TGA3 complex, thereby activating the expression of *PR* genes to enhance Arabidopsis resistance to diseases [253]. The salicylate hydroxylase gene *FgNahG* from *Fusarium graminearum* encodes a protein that can catalyze the conversion of SA to catechol in wheat against fusarium head blight. The expression of *FgNahG* in transgenic Arabidopsis reduces SA levels and decreases resistance to *F. graminearum* in leaves [254]. Furthermore, plant immune responses may involve crosstalk between different signaling pathways in the plants. Arabidopsis AtSR1 (a Ca^2+^/calmodulin-binding transcription factor) is a negative regulator of plant immunity, inhibiting the expression of *EDS1* (a SA regulator) by interacting with its promoter. In addition, the binding of Ca^2+^/calmodulin to AtSR1 is necessary for suppressing plant defense. Thus, the Ca^2+^ signal is linked to SA-mediated immune responses through calmodulin, AtSR1, and EDS1 [255]. Transcription factors play a crucial regulatory role in salicylic acid-mediated plant defense responses. Arabidopsis transcription factors TGA3 and WRKY53 interact with the CmYLCV promoter through the NPR1-dependent SA signaling pathway, enhancing its promoter activity [256]. 

Methyl salicylate (MeSA) plays a crucial role as a signaling chemical in regulating plant communication processes. In the natural environment, pathogen-induced volatile MeSA affects both the plant itself and neighboring plants. The expression of pathogenesis-related 1a (NbPR1a) and NbPR2, associated with disease resistance, is upregulated in response to MeSA treatment, confirming that MeSA enhances the systemic acquired resistance (SAR) capacity of tobacco. Thus, plants can perceive volatile defense chemicals, forming plant memory to enhance their SAR [257]. Indole, as a signaling chemical, is produced via the shikimate pathway via tryptophan or indole-3-pyruvate in higher plants. Indole can initiate the biosynthesis of defense-related metabolites and induce defense responses in tea trees infested with *Ectropis oblique* by enhancing Ca^2+^ signaling pathway and JA signaling [258]. Exposure of rice to exogenous indole induces the expression of its leucine-rich repeat receptor-like kinase gene (*OsLRR-RLK1*) and activates the expression of mitogen-activated protein kinases *OsMPK3* and *OsWRKY70* as well as JA biosynthesis-related genes, leading to the accumulation of JA, which regulates rice’s resistance to herbivores [259]. 

Jasmonic acid (JA) and methyl jasmonate (MeJA) are ubiquitous signaling chemicals in a plant’s response to biotic stressors. Feeding by herbivores induces the release of JA and MeJA from leaves, activating the expression of defense-related genes in neighboring healthy plants [113]. JA and MeJA are formed through the specific oxidation of C18 unsaturated fatty acids by lipoxygenase (LOX), resulting in intermediates with hydroperoxy groups at positions 9 and 13. The 13-hydroperoxide intermediate is then converted to JA through the catalysis of allene oxide synthase (AOS) and 12-oxo-phytodienoic acid reductase 3 (OPR3). JA is further transformed into methyl jasmonate (MeJA) under the catalysis of JA carboxyl methyltransferase (JMT). In plant cells, MeJA can be converted to jasmonic acid-isoleucine (JA-Ile) in an ATP-dependent manner by the action of jasmonic acid-amino synthetase (JAR1) [260]. 

Green leaf volatiles (GLVs) are produced in the fatty acid derivative pathway. The intermediate products of 9-hydroperoxide and 13-hydroperoxide are converted to C6 and C9 aldehydes through the action of hydroperoxide lyase (HPL). These aldehydes can then be reduced to alcohols and further transformed into esters. The resulting products are referred to as GLVs [261]. Z-3-hexenyl acetate (Z-3-HAC) in GLVs stimulates the generation of reactive oxygen species in wheat, leading to the regulation of the plant’s phenylpropanoid pathway and induction of its glycosylation reactions [106]. In Arabidopsis, after pre-treatment with (E)-2-hexenal, the content of anthocyanins significantly increases upon treatment with MeJA, indicating that (E)-2-hexenal can trigger MeJA-induced plant defense responses [107]. Z-3-hexenol (Z-3-HOL) can trigger a tomato plant’s defense pathways mediated by JA and SA, thereby enhancing resistance to whitefly infestation. Pre-treatment of maize with Z-3-HOL enhances the expression of the *ZmWRKY12* gene, which can regulate maize’s defense against herbivorous insects [108]. 

Allantoin plays an important role in plant stress and plant–organism interactions [120,189]. Root-secreted allantoin is also a potential signaling chemical for kin recognition in rice lines [87]. Allantoin is an intermediate product of purine metabolism. Adenosine monophosphate (AMP) and guanosine monophosphate (GMP) undergo deamination in the cytoplasm to form xanthine. Subsequently, xanthine is catalyzed by xanthine dehydrogenase (XDH) to produce urate. Urate then enters the peroxisome and is catalyzed by urate oxidase (UOX) to generate 5-hydroxyisourate (5-HIU). The 5-HIU is further converted to allantoin through the action of allantoin synthase [262]. Plants accumulate allantoin to help in scavenging reactive oxygen species and activating stress responses. The enhanced plant stress tolerance regulated by allantoin may be achieved through the activation of abscisic acid metabolism [263]. Allantoin can also activate the JA signaling pathway regulated by MYC2 through abscisic acid [264]. 

Biosynthesis and molecular mechanisms of signaling chemicals involve distinct biosynthetic pathways, signaling pathways, and regulatory transcription factors. However, plants are complex organisms; the reception of a chemical signal often triggers the crosstalk of several different signaling pathways. Therefore, a systematic analysis of the signal transduction pathways induced by plant chemical communication is essential to explore the key components and molecular mechanisms underlying signal crosstalk.

### 5.3. Molecular Mechanisms of Signaling-Induced Allelochemical Responses

**Figure 3:**
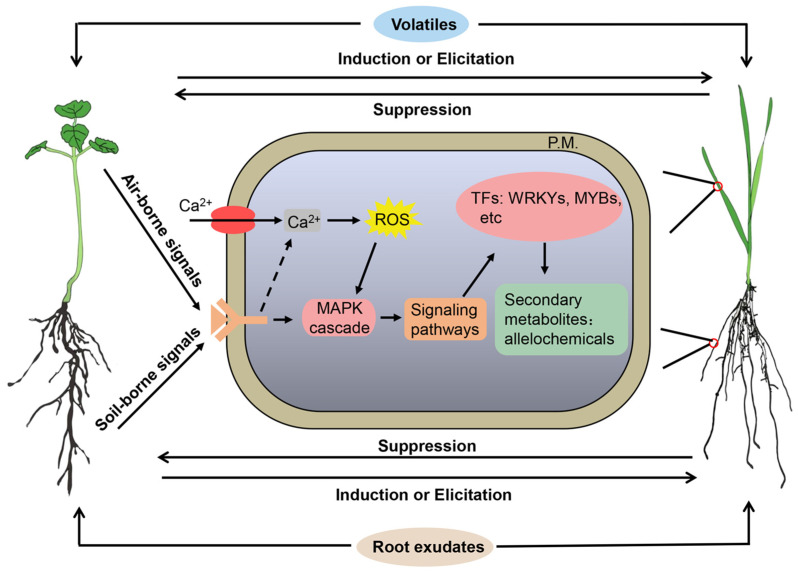
The signaling-induced allelochemical responses at the molecular level.

Plant neighbor detection and allelochemical response are driven by signaling chemicals. The signals released by neighbor plants enter the cell through stomata or the cuticle. After being recognized by receptors on the cell membrane, they enter the cell compartment (stomata or pass through the cuticular wax layer) and regulate changes in the plant cell’s ion flux, triggering a burst of ROS and activating MAPK and various signaling pathways. Subsequently, the activated pathways further regulate the expression of downstream genes and promote the synthesis of secondary metabolites, including allelochemicals and phytoalexins, etc. (Figure 3).

The cuticle serves as the final barrier for the release of VOCs into the atmosphere and acts as an obstacle for VOCs to enter the plant [265]. Previous research on stomata mostly focused on their involvement in plant immunity. Recent results indicate that stomata are also one of the effective pathways for VOCs to enter neighboring plants. During the day, VOCs can enter plants through stomata. At night, due to stomatal closure, signaling chemicals primarily diffuse through the cuticle layer for propagation [266]. The signals traverse the cell wall and bind to the receptor complexes on the membrane, thus further transmitting the signal downstream. However, there is limited research on signal receptors in plants [85]. The ethylene receptor ETHYLENE RESPONSE1 (ETR1) was identified in *Arabidopsis thaliana* in the 1990s [267]. Recently, TOPLESS-like proteins (TPLs) induced by THE tpl (*TOPLESS*) and tpr (*TOPLESS-related*) genes were found to specifically bind the signal β-ionone in tobacco. Simultaneously, they interact with TFs, modulating gene expression in hormone signaling (influencing auxin signaling) and stress responses (influencing JA signaling) [268]. Additionally, plants can facilitate the passage of VOCs through the membrane via transport proteins. After silencing the ABC transporter synthesis gene *PhABCG1* in *Petunia hybrida*, this protein is involved in the active transport of VOCs between plant cells [269]. Apart from traditional signals, plant signals can also be considered damage-associated molecular patterns (DAMPs), microbe-associated molecular patterns (MAMPs), and herbivore-associated molecular patterns (HAMPs) [270]. GLVs are fatty acid derivatives that may be sensed by leucine-rich repeat-receptor-like protein kinases (LRR-RLKs) localized on the membrane [266]. 

Calcium serves as a ‘second messenger’ involved in almost all external environmental responses and self-development signal transduction. Upon receiving external stimuli, there is a transient increase in calcium ion concentration in plant cells, leading to an influx. Under the action of Ca^2+^ sensors, such as CDPK, CaM, and CBL-CIPK, the signals are transmitted downstream [255]. Additionally, ocimene, myrcene, and pinene can also trigger cytosolic calcium influx in *Arabidopsis thaliana* [271]. ROS not only plays a role when plants are infected by pathogens but also is an indispensable part of the signal transduction network. The transcription factor *VqWRKY31* increases the accumulation of SA and ROS, facilitating the expression of defense-related genes and the accumulation of disease-resistant metabolites, including stilbenes, flavonoids, and proanthocyanidins, and enhances grapevine resistance to powdery mildew [272]. Furthermore, the production of ROS has been proven to depend on Ca^2+^ signals in many studies. After knocking out the Ca^2+^ channel genes *CNGC2* and *GLR3.3*, the ROS generation induced by MAMPs and DAMPs is significantly inhibited. In Arabidopsis, MAMP-induced ROS bursts are inhibited in *CDPK4/5/6/11* mutants, while the overexpression of *CDPK* promotes ROS generation in tomatoes [273]. A recent study has demonstrated that (–)-loliolide may mediate the content of allelochemicals based on Ca^2+^ and ROS signaling [132]. 

MAPK cascades are highly conserved and crucial signal transduction pathways in eukaryotes, primarily composed of three classes of protein kinases: MAPKKK/MEKK, MAPKK/MEK, and MAPK. MAPK cascades can receive upstream stimulus signals and amplify them through cascading, participating in the regulation of various processes such as plant growth, development, and responses to biotic and abiotic stresses [274]. Decreasing the expression of *MPK3* and *MPK4* can reduce the defense response induced by the volatile compound indole [259]. PD98059 is a MAPK inhibitor widely used in the research of MAPK cascades. After treating corn with PD98059, the activity of the ABA-mediated MAPK pathway was decreased and the expression of related antioxidant genes was downregulated. When grasses are cut or damaged, the released signal can rapidly activate the MAPK cascade response in neighbor plants, like *Lolium temulentum* [275]. Additionally, sesquiterpene (E)-nerolidol and indole can directly or indirectly regulate the expression of key genes in the MAPK cascade [259]. As a central pathway in plant metabolism, the MAPK cascade not only responds to upstream signals but also participates in regulating downstream hormone signal interaction networks and activating defense genes. A well-known example is the AtMAPK3/5-AtMAPKK4/5-AtMAPK3/6 cascade reaction, which is involved in regulating the synthesis of ETH and camalexin in Arabidopsis [276]. Furthermore, the MAPK cascades are also involved in the JA and SA signal cross-talk. *SlMPK6-1* and *SlMPK6-2* in tomatoes can act as positive regulators of JA biosynthesis and signal pathways, and silencing these genes can reduce the expression of JA biosynthesis and JA-dependent defense genes [277]. 

After the aforementioned series of signaling cascades, the synthesis of secondary metabolites, including allelochemicals, is regulated by activating corresponding transcription factors. TFs associated with secondary metabolites include WRKYs, MYBs, and others. For example, the transcription factor NaWRKY70 mediates the synergistic regulation of scopoletin and scopolin biosynthesis by JA and ethylene signaling [278]. The receptor proteins NPR1and NPR3/4, as well as the transcription factor TGA, are involved in the SA signaling pathway. The SmNPR4-SmNPR1-SmTGA2 and SmNPR4-SmTGA5 module mediates SA regulation of phenolic biosynthesis in *Salvia miltiorrhiza* [279]. MeJA upregulates *CYP98A14* by activating *SmMYB2* and *SmMYB98*, promoting the generation of phenolic substances [216]. Additionally, MeJA can also enhance the binding of *AaWRKY1* to the *ADS* promoter in *A. annua*, thereby increasing the content of artemisinin [280].

Plant–plant chemical interactions mediated by specialized metabolites are crucial for affecting plant performance and survival. Allelopathy and allelobiosis mediated by allelochemicals and signaling chemicals are interconnected and inseparable. There is extensive research on the signaling-inducing allelochemical response, but the molecular mechanisms involved remain limited. There is a lack of molecular evidence regarding how signaling chemicals affect the entire process of plant–plant allelopathic interactions. Therefore, the content introduced in this section primarily pertains to plant defense rather than solely allelopathic interactions. Exploring the signaling-induced allelochemical responses at the molecular level is crucial for a further understanding of the chemically mediated plant–plant interactions.

## 6. Perspectives and Outlooks

The importance of plant–plant interactions in fitness-determining processes, such as performance, survival, resource capture, and biochemical interactions, cannot be overemphasized in natural and managed ecosystems. Recent efforts have made considerable progress toward understanding allelopathy and allelobiosis in plant–plant interactions. Nevertheless, much work is needed to clarify the details of this interesting and important area in the near future. The central focus at present is the identification and determination of new signaling chemicals, particularly for soil-borne signaling chemicals. The others are as follows:

- Identifying chemical mediation before a plant–plant interaction is considered to be allelopathy or allelobiosis.
- Developing an ecological context for allelopathy and allelobiosis, particularly for a linkage between allelopathy and allelobiosis.
- Deciphering field-based evidence for allelopathy and allelobiosis in plant coexistence and community assembly.
- Detecting and determining spatiotemporal dynamics of allelochemicals and signaling chemicals from the living plants and their environments in situ.
- Interpreting the concept of plant neighbor detection and identity recognition, particularly for relatedness-mediated neighbor detection and identity recognition and their altering of the consequences of intra-specific and inter-specific interactions in species or cultivar mixtures.
- Deciphering root–soil interactions, particularly for chemical mechanisms that structure competitive rhizosphere interactions as well as the rhizosphere microbiome.
- Evaluating allelopathy and allelobiosis under environmental variability and global changes.
- Elucidating molecular mechanisms of allelopathy and allelobiosis in plant–plant interactions from gene expressions, protein receptors, and their transportation.
- Applying ecological approaches to agriculture and forestry by allelopathy with allelochemicals and allelobiosis with signaling chemicals.

Allelopathy and allelobiosis profoundly affect the performance of plants, altering the consequences of intra-specific and inter-specific interactions in plant coexistence and community assembly. Understanding the chemically mediated plant–plant interactions is necessarily a multidisciplinary science, integrating cellular processes, gene regulation, soil microbiology, and ecological interactions. Some new technologies, such as microdialysis, stable isotope labeling (e.g., ^13^C, ^15^N), imageology and multi-omics, and meta-transcriptomics, could shed light on identifying the role of allelopathy and allelobiosis in plant–plant interactions via the indirect way of rhizosphere microbiomes and belowground signaling interactions. In particular, recent methodological developments in high-resolution chemical detectives with molecular and imaging technologies will certainly clear the way for additional root chemical signals and their mediated interactions to be discovered. 

Allelopathy and allelobiosis synergistically impact interacting plants or coexisting species. Their effects may be expressed by changes in growth, competitive outcomes, and soil resource dynamics, among others. Of course, any effects and their consequences are only part of the potential suite of interactions between species or individuals within a species. It is the overall direction and strength of the interaction that will determine community dynamics and structure. What these more traditional ecological studies bring to the conceptual table is a broader perspective on the implications of chemically mediated plant–plant interactions and the potential to incorporate these interactions into broader theories of community organization. A thorough understanding of allelopathy and allelobiosis and the mechanisms underlying the chemically mediated plant–plant interactions not only broadens our insight into the eco-evolutionary relationships but also helps us to explore ecological approaches to improve tillage management in agriculture and forestry, and enhance the predictive ability of natural and managed ecosystems to respond to environmental changes.

## Figures and Tables

**Table 1:** Plant-released Aboveground and Belowground Signaling Chemicals.

	Signaling Chemicals	Function/Response	References
Air-borne signals	Ethylene	Alarm and induce defense	[102]
		Participate in plant neighbor detection	[116]
		Induce shade avoidance response	[117]
		Alarm for roots to avoid compacted soils	[118]
	Methyl jasmonate	Alarm and induce defense	[103]
		Attract natural enemies of the herbivores	[104]
	(*Z*)-3-Hexenyl acetate	Induce glycosylation reaction	[106]
	(*E*)-2-Hexenal	Induce defense responses	[107]
	(*Z*)-3-Hexenol	Induce defense responses	[108]
	β-caryophyllene	Induce defense responses	[109]
	Myrcene	Induce the accumulation of MeJA	[110]
	Ocimene	Induce the accumulation of MeJA	[110]
	4,8-Dimethylnona-1,3,7-triene	Induce defense responses	[111]
	(*E*)-Nerolidol	Induce defense responses	[112]
	4,8,12-Trimethyltrideca-1,3,7,11-tetraene	Induce defense responses	[113]
	Methyl salicylate	Induce defense responses	[105]
	Indole	Induce defense responses	[115]
	Methyl benzoate	Trigger phytotoxic effects	[114]
Soil-borne signals	Jasmonic acid	Induce allelopathy	[119]
		Participate in plant neighbor detection	[23]
	Salicylic acid	Participate in plant neighbor detection and allelopathy	[16]
	Allantoin	Participate in kin recognition in rice	[87]
		Activate stress response	[120]
	(–)-Loliolide	Participate in plant neighbor detection	[23]
		Induce defense responses	[121]
		Mediate plant kin recognition	[122]
		Modulate flowering time	[123]
		Modify the rhizosphere microbial community	[124]
Root–microbe signals	Strigolactones	The symbiosis between roots and arbuscular mycorrhizal fungi	[125]
		Induce parasitic weeds to search for potential hosts	[126]
	Luteolin Quercetin-3-*O*-rhamnoside	Attract rhizobia; induce the early formation of root nodules	[127]

## Data Availability

Not applicable.
